# Ultra-widefield swept-source OCT angiography assessment of choroidal changes in Chinese myopic adults

**DOI:** 10.3389/fmed.2026.1863719

**Published:** 2026-07-01

**Authors:** Chao Wu, Tianwei Liu, Fengjiao Li, Wanzhen Jiao, Bojun Zhao

**Affiliations:** 1 Department of Ophthalmology, Shandong Provincial Hospital Affiliated to Shandong First Medical University, Jinan, China; 2 Beijing Ophthalmology & Visual Sciences Key Laboratory, Beijing Tongren Eye Center, Beijing Tongren Hospital, Beijing Institute of Ophthalmology, Capital Medical University, Beijing, China; 3 Department of Ophthalmology, Jinan Second People's Hospital, Jinan, China

**Keywords:** axial length, choroidal thickness, ultra-widefield swept-source optical coherence tomography angiography, vessel density of the choriocapillaris, vessel density of the sattler and haller layer

## Abstract

**Purpose:**

To characterize region-specific choroidal alterations in Chinese young adults with varying refractive errors using ultra-widefield SS-OCTA (UWF SS-OCTA).

**Methods:**

In this cross-sectional study, 218 eyes from 109 participants underwent UWF SS-OCTA. Eyes were stratified by spherical equivalent (SE): low (LM), moderate (MM), and high myopia (HM). Choroidal thickness (CT), choriocapillaris vessel density (CCVD), and Sattler-Haller layer vessel density (SHVD) were quantified across nine subfields.

**Results:**

HM eyes showed significant CT reductions across all nine subfields and SHVD reductions in six subfields (all *p* < 0.05). Across groups, CT was thickest centrally and thinnest inferonasally; CCVD was lowest centrally; SHVD peaked superotemporally. CT correlated negatively with axial length (AL) in eight subfields and positively with SE in seven. SHVD correlated negatively with AL in seven subfields and positively with SE in six. CT and SHVD were positively correlated in six subfields. No significant associations were found for CCVD.

**Conclusion:**

Region-specific reductions in CT and SHVD occurred with increasing AL. Topographic patterns—central CT thickening, inferonasal thinning, central CCVD minima, and superotemporal SHVD maxima—were preserved across myopia severities. Both CT and SHVD showed significant negative correlations with AL.

## 1 Introduction

Myopia represents the most prevalent refractive disorder worldwide and constitutes a significant global public health challenge, with disproportionately high prevalence in Asian populations, particularly China (1, 2). Projections indicate that by 2050, approximately five billion individuals globally will be myopic, including one billion with high myopia (1–3). High myopia is characterized by excessive axial elongation, predisposing to sight-threatening complications including degenerative retinopathy, lacquer cracks, choroidal neovascularization, and posterior staphyloma (4, 5). These sequelae impose substantial socioeconomic burdens through irreversible visual impairment (6, 7). In recent years, growing studies have focused on the relationship between myopia and choroidal structure and function (8). These studies reveal significant microvascular and structural changes in high myopia compared with moderate myopia. High myopia is associated with distinctive alterations in the retina and choroid, including variations in retinal thickness, choroidal thickness, choroidal vascular index and avascular zone (9, 10).

Swept-source OCT (SS-OCT) offers superior imaging capabilities compared with spectral-domain OCT (SD-OCT), including faster scan rates, longer operational wavelengths (approximately 1,060 nm vs. 840 nm), reduced motion artifacts, and enhanced penetration depth, enabling improved visualization of the choroid and choroidal-scleral interface (11). BOptical coherence tomography angiography (OCTA) extends OCT technology by detecting signal decorrelation attributable to erythrocyte motion within perfused vasculature, thereby generating depth-resolved angiographic images (12). This technological synergy facilitates concurrent assessment of choroidal structural and vascular parameters. Prior SS-OCT investigations of the choroid were predominantly confined to small-field imaging of the macula or optic disc, limiting comprehensive topographic analysis (13–15). The advent of ultra-widefield SS-OCTA (UWF SS-OCTA) enables single-capture imaging extending anterior to the vortex vein ampullae in all four quadrants, permitting nuanced characterization of region-specific choroidal alterations in myopic eyes.

This study aimed to characterize spatial variations in choroidal thickness and vessel density and their associations with ocular biometric parameters using UWF SS-OCTA in Chinese young adults with myopia.

## 2 Materials and methods

### 2.1 Study design and participants

This cross-sectional observational study was conducted at Shandong Provincial Hospital Affiliated to Shandong First Medical University, Jinan, China, between March and September 2022. We enrolled 218 eyes from 109 graduate students recruited from Qilu Medical College, Shandong University.

Exclusion criteria comprised: (1) any ocular pathology detected on slit-lamp biomicroscopy or other ophthalmic examinations (except tessellated fundus), (2) best-corrected visual acuity (BCVA) worse than 20/20, (3) intraocular pressure (IOP) exceeding 21 mmHg, (4) history of ocular trauma or surgery, (5) history of ocular or systemic disease, and (6) smoking history. Eyes were stratified into three groups based on spherical equivalent (SE): low myopia (LM: -0.50 D > SE > −3.00 D; 80 eyes), moderate myopia (MM: -3.00 D ≥ SE > −6.00 D; 84 eyes), and high myopia (HM: SE ≤ −6.00 D; 54 eyes).

### 2.2 Ophthalmological examinations

All participants underwent comprehensive ophthalmic examinations, including BCVA assessment, cycloplegic refraction, slit-lamp biomicroscopy of the anterior segment, and dilated fundus examination. IOP was measured using an Icare rebound tonometer. Axial length was determined using optical low-coherence interferometry (IOL Master; Carl Zeiss Meditec, La Jolla, CA, USA). To minimize diurnal variations, participants were instructed to abstain from caffeine for 24 h and avoid fluid intake for 1 h prior to imaging. All measurements were performed between 20:00 and 22:00 h by a single trained examiner.

### 2.3 Image acquisition and analysis

UWF SS-OCTA imaging was performed using the BM-400 K system (TowardPi Medical Technology Co., Ltd., Beijing, China) operating at a center wavelength of 1,060 nm. The device achieved transverse and axial resolutions of 10 μm and 3.8 μm, respectively, with a scanning depth of 6 mm and an A-scan rate of 400,000 scans per second. The 24 × 20 mm angiography mode was employed. An integrated eye-tracking system based on confocal scanning laser ophthalmoscopy and proprietary motion-correction algorithms were utilized to minimize motion artifacts.

#### 2.3.1 Operational procedures

**Figure 1:**
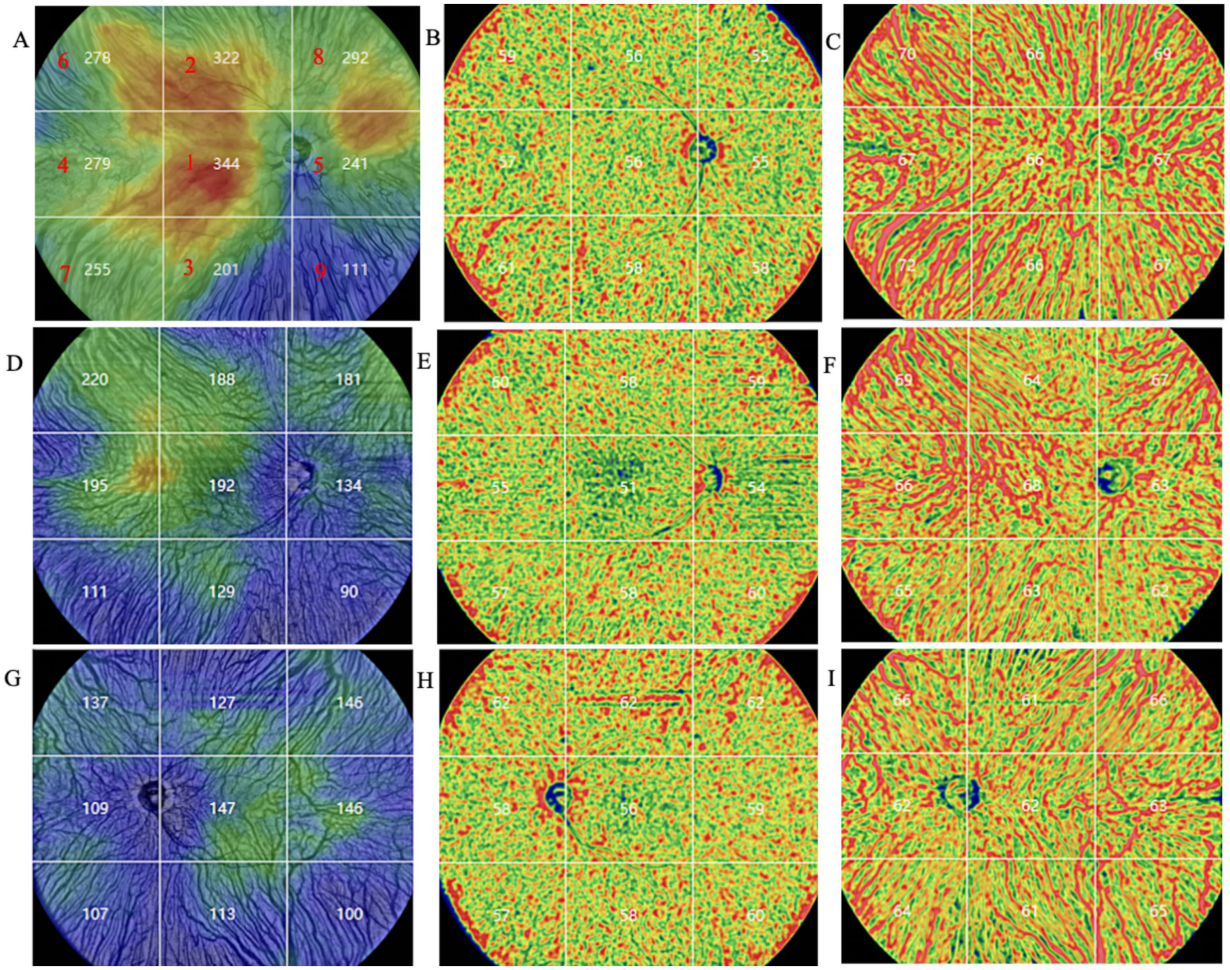
OCTA scans showing CT **(A)**, CCVD **(B)** and SHVD **(C)** in low myopia, CT **(D)**, CCVD **(E)** and SHVD **(F)** in moderate myopia,using color maps, CT **(G)**, CCVD **(H)** and SHVD **(I)** in high myopia using color maps. 1 to 9 labeled in picture **(A)** represents different 9 subfields.

The subject sits in front of the device and gazes straight ahead. The examiner manipulates the cross reticle on the screen with a mouse to align it with the center of the pupil and adjust the focal length properly. Instruct the subject to close eyes and stay still without moving, and keep eyes open steadily without blinking once scanning starts. After a 5-s rest, the subject opens eyes. Click the scan button. Keep the reticle centered on the pupil throughout acquisition, and follow pupil movement via mouse control to minimize image artifacts caused by unstable fixation and frequent blinking. Choroidal thickness (CT), choriocapillaris vessel density (CCVD), and Sattler and Haller layer vessel density (SHVD) were quantified using automated segmentation algorithms. The choriocapillaris slab was segmented from Bruch’s membrane to approximately 29 μm posteriorly. The Sattler and Haller layer slab extended from the choriocapillaris boundary to the choroidal-scleral interface. These parameters were automatically mapped onto a 3 × 3 grid comprising nine subfields (Figure 1): central (1), superior (2), inferior (3), temporal (4), nasal (5), superotemporal (6), inferotemporal (7), superonasal (8), and inferonasal (9). Images with motion artifacts, projection artifacts, or quality scores < 7 were excluded. All segmentations were manually verified and corrected when necessary prior to analysis. Axial length-related magnification errors were corrected using the modified Littmann formula (16).

### 2.4 Statistical analysis

The statistical analysis was performed by using SPSS software version 26 (IBM, ibm.com). Continuous variables were expressed in terms median and interquartile range (IQR). Classified data were reported as percentages. Then, Kruskal-Wallis test and Kruskal-Wallis univariate anova test were used to test the significance of difference among groups. Spearman’s correlation analysis was used to estimate relationships among the measurements. Significance was determined at *p* < 0.05.

## 3 Results

A total of 218 eyes from 109 participants were included. Demographic and clinical characteristics were comparable across the three myopia groups (Table 1).

**Table 1:** Demographics and characteristics of the study population (number, *n* = 218).

Parameters	LM	MM	HM	*p*
Patients, *n*	40	42	27	–
Eyes, *n*	80	84	54	–
Female, *n* (%)	20 (50)	16 (38)	12 (46)	0.544
Age (y), median (25th–75th percentile)	24 (22–26)	24 (22–26)	23 (22–27)	0.712
BCVA (logMAR), median (25th–75th percentile)	0 (0–0.1)	0 (0–0.1)	0 (0–0.1)	0.834
IOP (mmHg), median (25th–75th percentile)	16.2 (14.2–17.5)	15.9 (13.8–17.1)	16.4 (13.8–17.9)	0.621

### 3.1 Comparison of CT, CCVD and SHVD with different degrees of myopia

**Figure 2:**
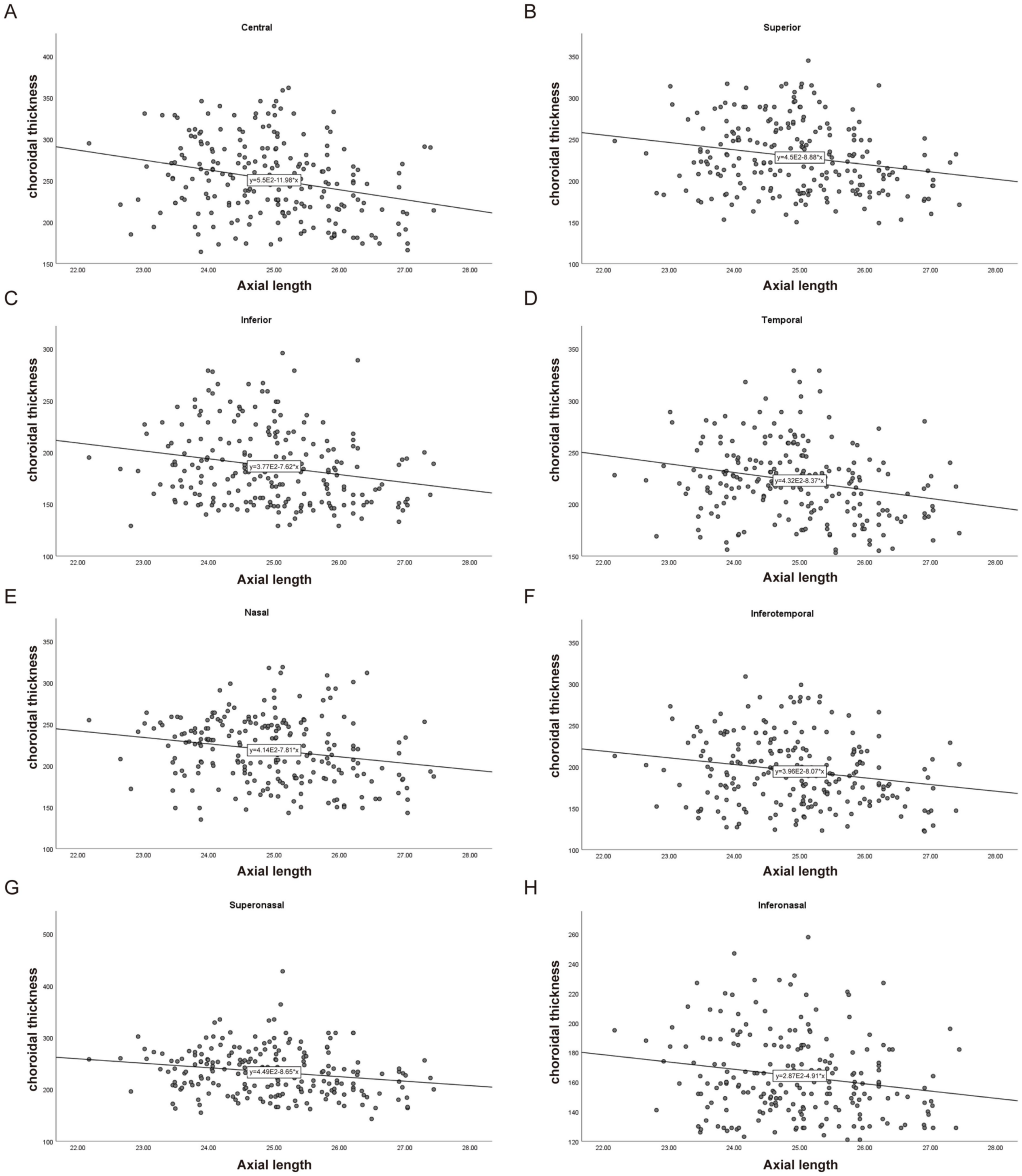
**(A–H)** Scatter plots of the changes of CT with AL from central to inferonasal areas. The linear regression equation formulas were shown in each plot.

Choroidal thickness differed significantly across myopia groups in most subfields (*p* < 0.01). *Post hoc* analysis revealed that the HM group exhibited significant reductions in CT (8 subfields) and SHVD (6 subfields) compared with the LM group (*p* < 0.05). Similarly, the HM group demonstrated decreased CT (4 subfields) and SHVD (2 subfields) relative to the MM group (*p* < 0.05). No significant differences were observed between the LM and MM groups for either CT or SHVD (*p* > 0.05). CCVD did not differ significantly among the three groups in any subfield (all *p* > 0.05; Table 2). Notably, CT reduction was most pronounced in the central subfield (Figure 2).

**Table 2:** Comparison of choroidal thickness and vascular metrics in eyes among different degrees of myopia.

Parameter	LM	MM	HM	*P*
CT (μm) by subfield, median (25th–75th percentile)				
Central (1)	252 (211–293)	264 (223–295)	241 (214–265)^a^	0.02
Superior (2)	223 (194–263)	237 (198–263)	211 (185–236)^a^	0.011
Inferior (3)	183 (158–221)	187 (159–217)	166 (151–190)^a^^,^^b^	0.012
Temporal (4)	224 (201–247)	227 (198–248)	210 (188–233)^a^^,^^b^	0.021
Nasal (5)	213 (184–249)	231 (203–253)	205 (184–247)	0.063
Superotemporal (6)	221 (191–251)	220 (189–241)	204 (178–229)^a^^,^^b^	0.048
Inferotemporal (7)	203 (175–234)	193 (159–228)	174 (157–199)^a^^,^^b^	0.004
Superonasal (8)	225(200–262)	246(210–271)	212(192–239)^a^	0.002
Inferonasal (9)	155(140–183)	167(152–193)	152(137–168)^a^	0.002
CCVD (%) by subfield, median (25th–75th percentile)				
Central (1)	55 (53–57)	55 (54–56)	55 (54–57)	0.982
Superior (2)	58 (57–60)	58 (57–60)	58 (57–61)^a^	0.04
Inferior (3)	58 (57–59)	58 (56–59)	58 (56–59)	0.881
Temporal (4)	56 (55–58)	56 (55–57)	56 (55–57)	0.732
Nasal (5)	58 (56–59)	57 (55–58)	58 (56–59)^b^	0.021
Superotemporal (6)	59 (58–60)	59 (58–60)	59 (57–61)	0.875
Inferotemporal (7)	58 (56–59)	57 (56–59)	59 (57–60)	0.126
Superonasal (8)	59 (56–60)	59 (56–60)	59 (58–60)	0.147
Inferonasal (9)	59 (57–60)	59 (58–60)	60 (59–61)	0.091
SHVD (%) by subfield, median (25th–75th percentile)				
Central (1)	64 (63–65)	64 (63–65)	63 (63–64)	0.056
Superior (2)	63 (62–64)	63 (62–64)	62 (61–63)^a^	0.019
Inferior (3)	64 (62–64)	63 (62–64)	63 (62–64)	0.093
Temporal (4)	65 (64–65)	64 (64–65)	64 (63–65)^a^	0.001
Nasal (5)	64 (63–65)	64 (63–65)	64 (62–65)	0.482
Superotemporal (6)	67 (66–68)	67 (66–68)	66 (65–67)^a^	0.048
Inferotemporal (7)	67 (66–69)	67 (65–68)	65 (64–67)^a^^,^^b^	<0.001
Superonasal (8)	67 (66–67)	66 (65–67)	66 (65–67)^a^	0.025
Inferonasal (9)	65 (63–66)	65 (63–66)	63 (62–65)^a^^,^^b^	0.001

*p* values meant the significance of comparisons among three groups.

a Compared with LM group, there was a significant decrease in HM group (*p* < 0.05).

b Compared with MM group, there was a significant decrease in HM group (*p* < 0.05).

### 3.2 Comparison of CT, CCVD and SHVD with different subfields in each myopic group

**Figure 3:**
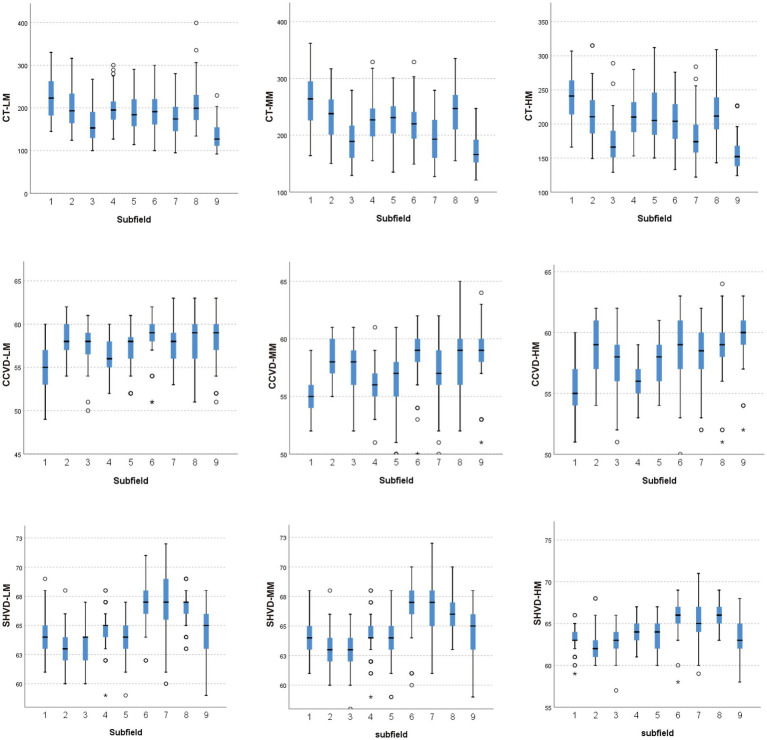
**(A–C)** Boxplots of CT of different grids in each myopic subgroup. One to 9 represents 9 subfields, respectively. **(D–F)** Boxplots of CCVD of different grids in each myopic subgroup. **(G–I)** Boxplots of SHVD of different grids in each myopic subgroup.

Within each myopia group, CT, CCVD, and SHVD exhibited significant topographic variations (all *p* < 0.01). Pairwise comparisons among subfields revealed consistent patterns across groups: CT was maximal centrally (thicker than 6 other subfields; *p* < 0.05) and minimal in the inferonasal region (thinner than 8 other subfields; *p* < 0.05). CCVD was lowest in the central subfield (higher than 7 other subfields; *p* < 0.05). SHVD was highest in the superotemporal region (higher than 6 other subfields; *p* < 0.05) and lowest inferiorly (lower than 7 other subfields; *p* < 0.05) (Table 3; Figure 3).

**Table 3:** Significance of pairwise comparison of CT and vascular metrics among different subfields with same myopia group.

Subfield^a^	CT			Subfield^a^	CCVD			Subfield^a^	SHVD		
	LM	MM	HM		LM	MM	HM		LM	MM	HM
9 & 8	< 0.001	< 0.001	< 0.001	1 & 9	< 0.001	< 0.001	0.04	3 & 9	< 0.001	< 0.001	0.08
9 & 7	0.005	< 0.001	0.08	1 & 8	< 0.001	< 0.001	< 0.001	3 & 8	< 0.001	< 0.001	< 0.001
9 & 6	< 0.001	< 0.001	< 0.001	1 & 7	< 0.001	< 0.001	< 0.001	3 & 7	< 0.001	< 0.001	< 0.001
9 & 5	< 0.001	< 0.001	< 0.001	1 & 6	< 0.001	< 0.001	< 0.001	3 & 6	< 0.001	< 0.001	< 0.001
9 & 4	< 0.001	< 0.001	< 0.001	1 & 5	< 0.001	0.036	0.002	3 & 5	0.09	0.008	0.032
9 & 3	0.08	0.009	0.06	1 & 3	< 0.001	< 0.001	0.005	3 & 4	< 0.001	< 0.001	0.001
9 & 2	< 0.001	< 0.001	< 0.001	1 & 2	< 0.001	< 0.001	< 0.001	3 & 1	0.08	0.022	0.07
1 & 9	< 0.001	< 0.001	< 0.001	4 & 9	< 0.001	< 0.001	< 0.001	6 & 9	< 0.001	< 0.001	0.001
1 & 7	< 0.001	0.015	< 0.001	4 & 8	< 0.001	< 0.001	< 0.001	6 & 5	< 0.001	< 0.001	0.008
1 & 6	< 0.001	0.002	0.04	4 & 7	0.001	0.007	0.001	6 & 4	< 0.001	< 0.001	0.04
1 & 5	0.006	< 0.001	0.04	4 & 6	< 0.001	< 0.001	< 0.001	6 & 2	< 0.001	< 0.001	< 0.001
1 & 4	0.005	0.08	0.12	4 & 3	0.009	< 0.001	0.08	6 & 1	< 0.001	< 0.001	< 0.001
1 & 3	< 0.001	< 0.001	< 0.001	4 & 2	< 0.001	< 0.001	< 0.001	3 & 4	0.002	0.004	0.041

a Two subfields where parameters compared with each other, and from 1 to 9 represents central, superior, inferior, temporal, nasal, superotemporal, inferotemporal, superonasal and inferonasal area, respectively.

### 3.3 Correlations among CT, CCVD and SHVD

**Figure 4:**
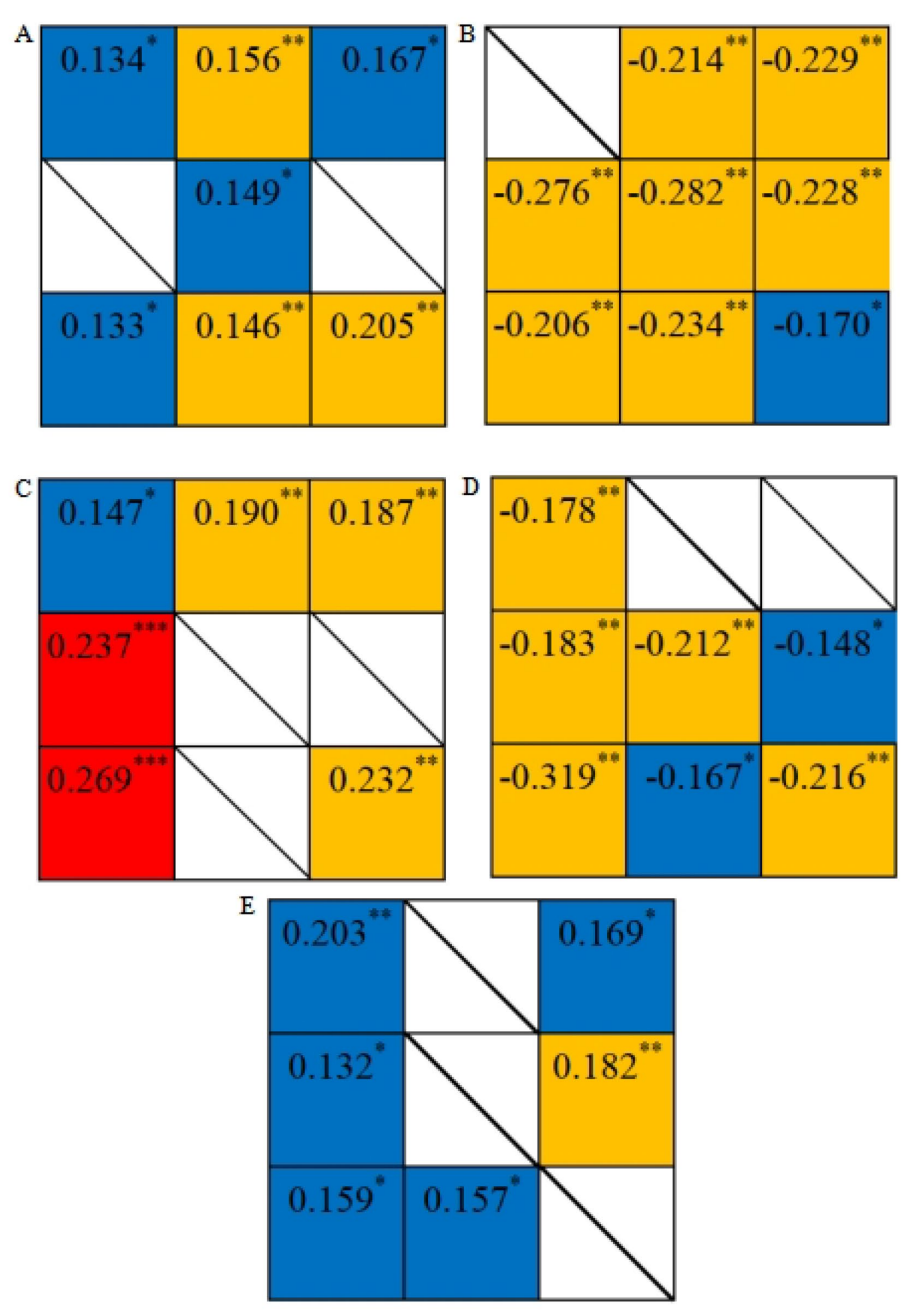
**(A)** Correlations of CT and SE. **(B)** Correlations of CT and AL. **(C)** Correlations of SHVD and SE. **(D)** Correlations of SHVD and AL. **(E)** Correlations of CT and SHVD. The correlation coefficient was represented as the value in 9 subfields. ∗∗∗*p* < 0.05, in red; ∗∗*p* < 0.01, in yellow; ∗*p* < 0.001, in blue.

Spearman’s rank correlation analysis revealed significant negative associations between CT and AL in seven subfields (*p* < 0.05). Similarly, CT correlated positively with SE in seven subfields, with the exception of the nasal and temporal regions. SHVD demonstrated negative correlations with AL in seven subfields and positive correlations with SE in six subfields (all p < 0.05). Additionally, CT and SHVD were positively correlated in six subfields (p < 0.05). In contrast, no significant correlations were observed between choriocapillaris vessel density (CCVD) and CT, AL, or SE (*p* > 0.05) (Figure 4).

## 4 Discussion

This study employed UWF SS-OCTA to comprehensively characterize choroidal structural and microvascular parameters in young Chinese adults with myopia. Key findings include: (1) significant reductions in CT (particularly centrally) and SHVD in high myopia; (2) preserved topographic patterns across myopia severity groups, with central CT thickening, inferonasal thinning, central CCVD minima, and superotemporal SHVD maxima; (3) negative correlations of CT and SHVD with AL; (4) positive correlations between CT and SHVD; and (5) lack of association between CCVD and axial elongation.

Spaide’s description of the enhanced depth imaging (EDI) protocol for SD-OCT represented a milestone in noninvasive *in vivo* choroidal imaging (17). However, EDI necessitates multiple scan averaging to achieve adequate contrast and signal-to-noise ratios, precluding simultaneous optimized visualization of the vitreous, retina, and choroid. Furthermore, the shorter wavelength of SD-OCT (840 nm) often precludes adequate penetration to reliably identify the choroidal-scleral interface, frequently requiring manual segmentation (18). SS-OCTA circumvents these limitations through its longer operating wavelength (1,060 nm), faster acquisition speeds, and automated segmentation capabilities. Notably, recent histopathological correlation studies have demonstrated that UWF SS-OCTA provides CCVD and SHVD assessments comparable to ex vivo tissue analysis (19). Moreover, the UWF SS-OCTA system employed herein has demonstrated excellent reproducibility for CT, CCVD, and SHVD quantification (19).

Our findings align with previous reports demonstrating significant CT reductions in high myopia (20, 21). We observed negative correlations between CT and AL in eight subfields, consistent with the well-established inverse relationship between axial elongation and choroidal thickness (22, 23). Notably, central choroidal thinning was most pronounced in high myopia, corroborating prior SS-OCTA investigations (15, 19).

In high myopia, SHVD was significantly reduced in six subfields, showing negative correlations with axial length (AL) and positive correlations with spherical equivalent (SE). This aligns with Devarajan et al. (21), who reported reduced medium-to-large choroidal vessel density in high myopia, likely due to axial elongation-induced vascular atrophy and stretching (24). The attenuated SHVD–AL relationship between moderate and high myopia suggests a threshold effect, whereby notable vascular rarefaction occurs only beyond a certain degree of axial elongation, consistent with the more pronounced choroidal thinning reported by Flores-Moreno et al. (23) No significant differences in CCVD, nor correlations between CCVD and AL or SE, were observed among the three myopia groups (*p* > 0.05), consistent with prior studies (19, 25, 26). However, Cheng et al. (27) found that choriocapillaris flow deficit percentage (CC FD%) increased with longer AL in non-pathological high myopia within the perifoveal region (6 × 6 mm macular imaging), where a higher CC FD% indicates lower choriocapillaris density. Methodological differences may explain the discrepancy: Weijing et al. used a 10 μm slab from the retinal pigment epithelium–Bruch’s membrane, whereas our device automatically segmented the choriocapillaris from Bruch’s membrane to 29 μm below it. Additionally, the high myopia eyes in their study were more severely affected, and their participants were aged 30–80 years, compared to 20–29 years in our cohort.

We observed consistent topographic patterns across all myopia groups: maximal central CT and minimal inferonasal CT. This aligns with Tanabe et al. (28) observation of physiologically thinner choroid inferonasal to the optic disc, a region potentially vulnerable to hypoxia and elevated IOP. Yazdani et al. (29) found that the choroid (in cross-line scanning up to 14 mm) was thickest superiorly, and thinnest nasally and peripheral choroidal thinning was greatest in the nasal quadrant and smallest in the superior quadrant. Despite different ranges and subfields, studies found that the thinner choroidal areas tended to spread nasally and inferiorly, which was consistent with our study. Though the mechanisms are still unknown.

We found that CCVD tended to be minimum in the central region. Nassisi et al. (30) reported that the CC FD% tended to decrease from the fovea towards the periphery (in 6 × 6 mm pattern). Ramrattan et al. (31) demonstrated that CCVD decreases with age together with a significant increase in Bruch’s membrane (BM) thickness. Nassisi et al. (30) speculated that the thickening of BM was more prominent under the fovea, leading to an increased drop off on OCTA at the level of the choriocapillaris, hence to an overestimation of the flow deficits in this zone. Besides, Nassisi et al. (30) proposed that compared with other areas, the CC under the fovea could be subjected to more stress to dispose the metabolites produced by the overlying RPE and photoreceptors, leading to a faster impairment of the blood microcirculation under the fovea. Therefore, it is possible that after a point, the CC impairment under the fovea could lead to some pathologies, such as atrophy, neovascularization, age-related macular degeneration, etc. CCVD could be a potential biomarker for monitoring possible abnormalities, especially with the coming of SS-OCT era which has made a more accurate quantification of CCVD possible. We found that SHVD tended to be maximum in the superotemporal subfield. Besides, SHVD in inferotemporal, superonasal and inferonasal areas were higher than other regions, which may correlate with the existing of the vortex ampulla. We found that thinning CT correlated with decreased SHVD in many subfields. However, faced the results that CT tended to be thickest in the central area and SHVD tended to be maximum in the superotemporal area, there must be some other factors at work.

In our study, CCVD did not vary significantly among the three groups in any subfield. This negative finding was consistently observed across all nine subfields. This lack of difference may be attributable to the fact that our study cohort comprised young, healthy individuals without myopic fundus pathologcal change., Furthermore, in the early to intermediate stages of myopia, the choriocapillaris may exhibit a compensatory increase to satisfy the metabolic requirements of the outer retina, thus offsetting any potential reduction in vessel density that could otherwise result from mechanical stretch. This is consistent with previous studies (19, 25, 26). Meanwhile,we found CT and SHVD were positively correlated in six subfields,our analysis suggests that this might be attributable to the Sattler and Haller layers comprise the bulk of the choroidal volume, making their vascular health the principal determinant of overall CT. Our finding that axial length significantly affected SHVD in young adults, whereas choriocapillaris vessel density showed no significant differences, supports this, as it confirms that deep choroidal layers are the key structural drivers of myopia-induced changes. Future studies incorporating different age groups and diverse myopic populations are needed to validate and extend our current observations.

There are some limitations. First, only Chinese participants aged 22 to 29 years were included in our study. The findings of this study may not be directly applicable to other ethnic groups. And it is unclear whether the results can be applied to other Chinese age groups. Second, in this study, both eyes of 109 participants were included,we acknowledge that selecting only one eye per individual is preferable to minimize confounding bias derived from interocular correlation. We will conduct further exploration with an expanded single-eye sample size in subsequent research. Third, emmetropic eyes were not included in our study, which may lead to an inaccurate and insignificant results. The sample size involved in our study was small, especially to the HM group’s, which may bias the results. We also consider that the inclusion of an emmetropic group would provide a baseline reference. Future studies with larger sample sizes are needed. Fourth, due to the influence of sample sizes across the myopia groups, our results did not include an assessment of group differences in the correlations of CT, CCVD, and SHVD. We plan to address this limitation in future studies by enrolling a larger sample size to further validate our findings. Fifth, due to the limitation of cross-sectional study, causal inferences about these findings cannot be made. Further longitudinal studies are needed. Finally, manual adjustments were required in some cases to correct the inaccurate segmentation, which may associate with errors.

In conclusion, this study found that a more comprehensive features of choroid could be quantified by UWF SS-OCTA. CT tended to be thickest in the central region and thinnest in the inferonasal area. CCVD tended to be minimum in the central region. SHVD tended to be maximum in the superotemporal area and minimum in the inferior region. CT (especially in the central area) and SHVD were negatively correlated with AL in most subfields. These findings enhance understanding of myopia-related choroidal pathophysiology and may inform future therapeutic strategies targeting choroidal remodeling.

## Data Availability

The original contributions presented in the study are included in the article/supplementary material, further inquiries can be directed to the corresponding author.
